# The Role of Regional Anesthesia During the SARS-CoV2 Pandemic: Appraisal of Clinical, Pharmacological and Organizational Aspects

**DOI:** 10.3389/fphar.2021.574091

**Published:** 2021-06-04

**Authors:** Gianluca Cappelleri, Andrea Fanelli, Daniela Ghisi, Gianluca Russo, Antonio Giorgi, Vito Torrano, Giuliano Lo Bianco, Salvatore Salomone, Roberto Fumagalli

**Affiliations:** ^1^ Anesthesia and Intensive Care Unit, Policlinico di Monza, Monza, Italy; ^2^ Anesthesia, Postoperative Intensive Care and Pain Therapy, Rizzoli Orthopedic Institute (IRCCS), Bologna, Italy; ^3^ Anesthesia, Postoperative Intensive Care and Pain Therapy, Lodi Hospital, Lodi, Italy; ^4^ Department of Medicine and Surgery, University of Milano Bicocca, Monza, Italy; ^5^ Department of Anesthesia, Critical Care and Pain Medicine, Asst Grande Ospedale Metropolitano Niguarda, Milan, Italy; ^6^ Department of Biomedical and Biotechnological Sciences, School of Medicine, University of Catania, Catania, Italy; ^7^ Anesthesiology and Pain Department, Fondazione Istituto G.Giglio, Cefalú, Italy; ^8^ Department of Anesthesia, Critical Care and Pain Medicine, Niguarda Ca' Granda Hospital, Milan, Italy; ^9^ University of Milano-Bicocca, Milan, Italy

**Keywords:** COVID-19, regional anaesthesia, severe acute respiratory syndrome coronavirus 2, coronavirus, anesthesia, conduction, regional anesthesia

## Abstract

The severe acute respiratory syndrome coronavirus SARS-CoV2 is spreading over millions of people worldwide, leading to thousands of deaths, even among the healthcare providers. Italy has registered the deaths of 337 physicians and more than 200 nurses as of March 14, 2021. Anesthesiologists are at higher risk as they are the care providers in both ICU and operating rooms.Although the vaccination of healthcare providers has been the prioirity, physicians are still continually exposed to the virus and potentially risk contagion and must thus protect themselves and their patients from the risks of infection while providing the best care to their surgical patients.Regional anesthesia allows for a reduction in airway manipulation, reducing environmental contamination as a result. Furthermore, regional anesthesia reduces the opioid requirements as well as the muscle paralysis due to muscle-relaxants and should be recommended whenever possible in COVID-19 patients. Our aim is to evaluate the advantages and criticisms of regional anesthesia in the management of surgical patients in the pandemic age.

## Introduction

For over a year, the severe acute respiratory syndrome coronavirus SARS-CoV2 has posed a challenge to many health systems worldwide, leading to thousands of deaths, even among the healthcare providers. Italy alone has registered more than 100,000 deaths, including 337 physicians and more than 200 nurses, as of March 14, 2021 (IstitutoSuperiore di Sanità, 2021).

**FIGURE 1:**
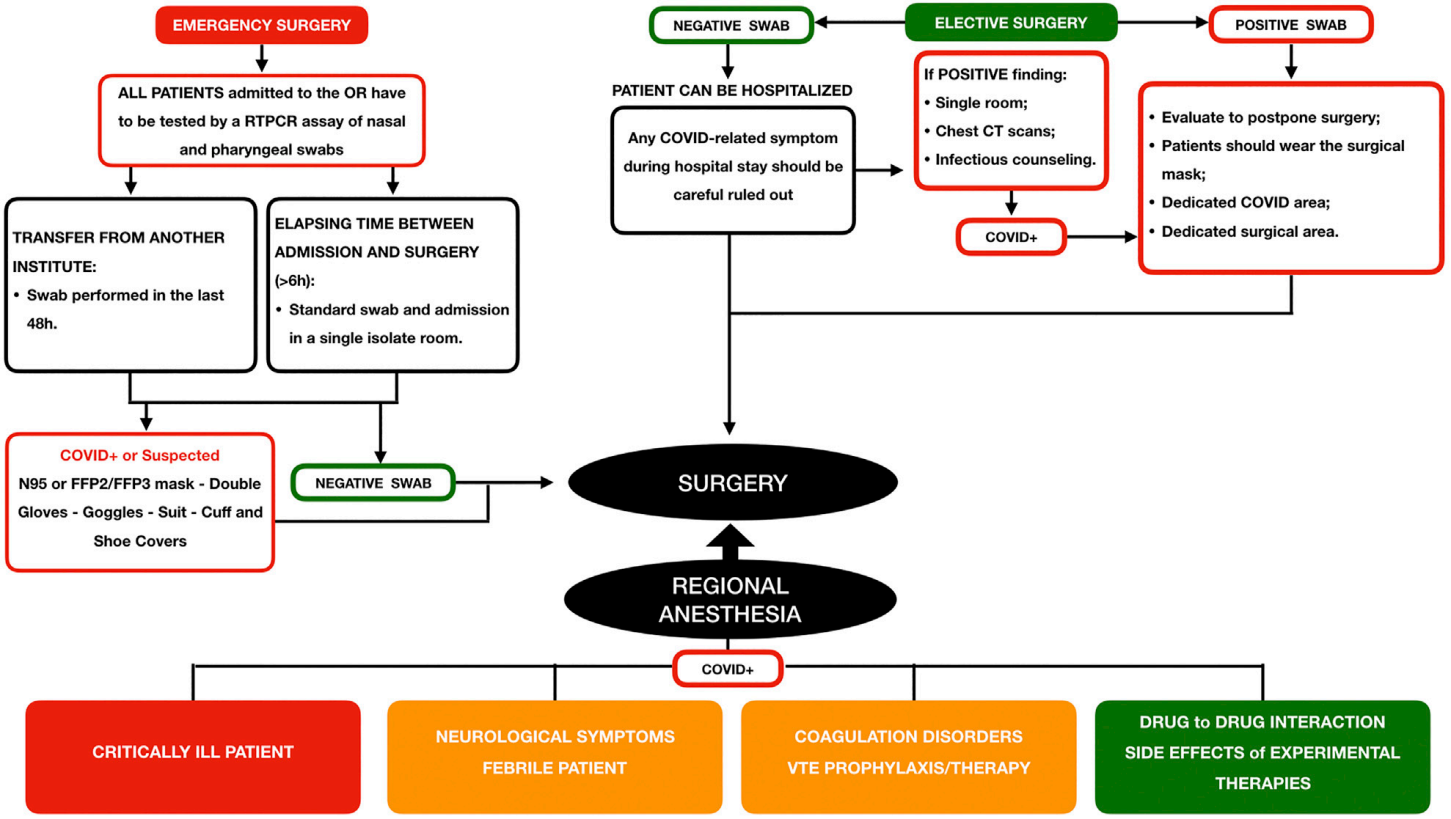
Phase two of the COVID-19 pandemic: surgical patient’s management protocol and the role of regional anesthesia (adapted by “ASST Grande Ospedale Metropolitano”. Niguarda Hospital, https://accessoweb.ospedaleniguarda.it:11050/img/upload/files/2836_attivit__chirurigca.pdf).

After the first lockdown that followed the surge of COVID-19 infection in late February 2020, only a certain percentage of elective surgery has continued. This situation has led to inadvertent close contact between COVID patients and COVID-free patients, increasing the risk of spreading. By considering that approximately 80% of infected individuals present with no or only mild symptoms of respiratory infection (Landau et al., 2020), the main organizational challenge still remains the early recognition of all infected patients before admission to the operating rooms (Figure 1). Particularly, in case of COVID-free surgery, the main purpose is to protect COVID-negative patients from COVID-positive patients and possibly infected workers. This latter occurrence is of the utmost importance since the recent introduction of the vaccines seems to protect from the disease but does not necessarily hamper the spread of the infection (Bhopal et al., 2021). Yet, the postoperative pathway may become a main concern. Longer hospital stays could increase the risk of infection for patients with a reduced immune response due to surgery.

During the last pandemic year, a number of major hospitals (Hubs) have performed both elective COVID-free surgery and all the non-deferrable surgeries in COVID positive patients. “ASST Grande Ospedale Metropolitano,” Niguarda hospital in Milan, has been identified as a regional hub for major trauma and neurosurgical emergencies as well as for COVID patients. The coexistence of entire floors dedicated to COVID-19-infected patients together with COVID-free patients present for elective or urgent surgical indications in the same hospital has put the healthcare system under great pressure, forcing a revision of the surgical pathway.

From March 1 to December 31, 2020, in the emergency department alone, a total of 313 surgical procedures were performed on patients with a real-time reverse transcriptase-polymerase chain reaction (RTPCR) assay of nasal and pharyngeal swabs that were positive for COVID-19 infection (175 orthopedic, 35 neurosurgical, 9 maxillo-facial, 25 general surgery, and 69 plastic surgery). Regional anesthesia (RA) was performed on 170 (97%) of all the orthopedic patients and on 43 (30%) of the remaining surgical patients. None of them have shown complications related to the RA technique even in mild symptomatic patients.

At the moment, the regional government of Lombardy stated that surgical procedures have to be limited to 60–70% of the usual activity in order to ensure sufficient hospital accesses is available for COVID-19-infected patients. With these expectations, a reduction in the length of hospital stay for COVID-free patients represents one of the main issues. RA has proven to be effective with this purpose (Joshi and Kehlet, 2019). The advantages of reducing the length of hospital stay of RA are 1) The opioid-sparing effects, 2) The improvement of both respiratory and the bowel functions, 3) Overall reduction of the side effects, and 4) Better postoperative analgesia (Jakobsson and Johnson, 2016; Capdevila et al., 2017).

Additionally, RA offers further advantages in case of confirmed or only suspected COVID-19 patients. It limits the airway manipulation, reducing the exposure to patient’s respiratory droplets and the risk of spreading the virus in operating rooms; as a result, both the American Society of Regional Anesthesia and Pain Medicine (ASRA) and the European Society of Regional Anesthesia and Pain Therapy (ESRA) have recommend the use of RA whenever possible in COVID-19 infected patients (American Society of Regional Anesthesia and Pain Medicine, 2020).

In spite of these potential advantages, we believe that the role of RA in the age of the pandemic should be carefully weighed. In the selection of a proper anesthesia technique, the anesthesiologists should also take into account the safety of RA related to the clinical presentation of SARS-CoV2, the advantages in terms of the drug-to-drug interactions and side effects of experimental therapies for SARS-CoV2, and the safety of RA with regards to the coagulation disorders showed by COVID patients.

## Regional Anesthesia and Clinical Manifestation of COVID-19 Disease

Based on the few data available related to the safety of RA in COVID-positive patients, and starting from the characteristics of the SARS-CoV2 infection, both neuraxial techniques, and peripheral nerve blocks seem to be safe during all stages of the disease except in the critically ill patient. RA should be considered in particular when the entire procedure can be done solely under RA (Stundner and Memtsoudis, 2012; Ashokka et al., 2020). Careful preoperative risk stratification should be conducted in patients with a moderate to severe pulmonary presentation of COVID 19: excessive cough, inability to lie in supine position, and/or dyspnea; these should be considered potential indications for general anesthesia.

The potential advantages of RA over general anesthesia (GA) have been reported by a review evaluating the obstetric anesthesia management during the COVID-19 pandemic (Bauer et al., 2020). Despite the small number of data available on COVID-19 infection, the authors confirmed that neuraxial labour analgesia remains a mainstay of obstetric care by limiting the exacerbation of respiratory symptoms associated with labour pain and the need for general anesthesia in case of intrapartum caesarean delivery. (Zhong et al., 2020). evaluating the outcome of 49 patients undergoing surgery (mainly for caesarean section, 92%), in whom spinal anesthesia (SA) was undertaken, confirmed that SA appears to be safe in mild symptomatic COVID-19 patients (Zhong et al., 2020). Chen et al. (2020) reported on the safety and the efficacy of different anesthetic regimens in 17 parturients with COVID-19 undergoing caesarean delivery. The majority of the patients (82%) received RA for caesarean section, and only three patients (18%) received GA. In this study population, four patients (24%) presented with mild fever without chills, four patients (24%) with cough, and two patients (12%) with chest distress. Fatigue, dyspnea, and diarrhea were the symptoms of the other three patients. All the symptomatic patients received a continuous epidural anesthesia. The authors reported that hypotension (<30% reduction from baseline) occurred in 12 out of 14 cases with epidural anesthesia. Perioperative hypotension was effectively treated using a combination of left lateral position, intravenous fluids, and vasopressor support (phenylephrine) (Chen et al., 2020).

Careful attention should be paid to patients with neurological symptoms related to COVID-19 infections such as headaches, consciousness disorder, paresthesia, and other pathological signs that could potentially interfere with RA follow-up (Wu et al., 2020).

To date, there are no well-established guidelines for the anesthetists in the choice of anesthesia for febrile patients, particularly if the fever shows a viral etiology. Severe central neuraxial infections, such as arachnoiditis, meningitis, and abscess after spinal or epidural anesthesia, are rare (Wedel and Horlocker, 2006). As reported by Ashokka et al. (2020) in a systematic review related to RA in the critically ill patient, neuraxial block can be considered in patients with a white cell count < 15 × 10^9/L who also have a left ventricular ejection fraction higher than 35% and stabilized systemic blood pressure. In this fragile population, peripheral nerve blocks provide effective anesthesia in patients undergoing surgery on the extremities or chest wall with negligible cardiovascular impact.

Finally, in a large multicenter study including 1128 patients in 235 hospitals with SARS-CoV-2 infection confirmed within 7 days before or 30 days after surgery, pulmonary complications occurred in 577 (51.2%) patients; the 30-day mortality in these patients was 38% (219 of 577), accounting for 81.7% (219 of 268) of all deaths (COVIDSurg Collaborative*, 2020). Also taking into account these further risks, the decision to perform regional anesthesia must be made on an individual basis, considering the anesthetic alternatives, the benefits of RA, and the risk of central nervous system infection that may theoretically occur in case of untreated systemic infection (Wedel and Horlocker, 2006).

## Anesthesia Interactions with the Experimental COVID-19 Therapy

The specific risk of drug-to-drug interactions and side effects of COVID-19 medications have to be considered during the choice of the anesthesia technique. In the last year several drugs had been investigated for the COVID-19 management such as the associations of lopinavir/ritonavir, darunavir/cobicistat, emapalumab/anakinra, as well as the antimalarial agent’s chloroquine and hydroxychloroquine. Most of them did not turn out to be effective in the COVID-19 treatment and were even harmful in some cases (Borba et al., 2020). As of March 22, 2021, the main agents currently in use, or under investigation, are azithromycin, low molecular weight heparin (LMWH), dexamethasone, and remdesivir (The Liverpool Drug Intera, 2020). The potential risk to drug-to-drug interaction has been evaluated for medications routinely used in the anesthesia practice, including local anesthetics (bupivacaine, etidocaine, and lidocaine), sedative-hypnotic agents (dexmetedomidine, propofol, and sevoflurane), muscle relaxants (rocuronium), and opioids (alfentanyl, buprenorphine, fentanest, methadone, morphine, and sufentanil) (The Liverpool Drug Intera, 2020; Table 1).

**TABLE 1:** A summary of possible drug–drug interactions between the experimantal COVID-19 therapies and the main anesthesia-related medications.

	ATV	LPV/r	RDV	RBV	TCZ	FAVI	CLQ	HCLQ
Bupivacaine	↑	↑	=	=	↓	=	=	=
Dexmetedomidine	=	↓	=	=	=	=	=	=
Etidocaine	↑	↑	=	=	↓	=	=	=
Ketamine	↑	↑	=	=	↓	=	=	=
Propofol	=	↓	=	=	=	=	=	=
Rocuronio	↑	↑	=	=	=	=	↑	↑
Sevoflurane	=	=	=	=	=	=	=	=
Sufentanil	↑	↑	=	=	↓	=	=	=
Alfentanil	↑	↑	=	=	↓	=	=	=
Buprenorphine	↑	↑2%	=	=	↓	=	=	=
Codeine	=	↑	=	=	=	=	↑	↑
Fenatanyl	↑	↑	=	=	↓	=	=	=
Hydrocodone	↑↓	↑↓	=	=	=	=	↑	↑
Methadone		↓53%	=	=	=	=	=	=
Morphine		↓	=	=	=	=	=	=
Oxycodone	↑	↑	=	=	↓	=	=	↑
Paracetamol	=	=	=	=	=	↑14−16%	=	=
Tramadol	↑	↑	=	=	=	=	=	=

ATV = Atazanavir, LPV/r = Lopinavir/ritonavir, RDV = Remdesivir, RBV = Ribavirin, TCZ = Tolicizumab, FAVI = Favipiravir, Chloroquine, HCLQ = Hydroxychloroquine.

↑Potential increased exposure of the comedication

↓Potential decreased exposure of the comedication

=No significant effect

The main concern relates to the use of opioid analgesics in patients with concomitant antiviral therapy. As for ritonavir, this class of agents may cause profound inhibition of hepatic and first-pass CYP3A activity (Kharasch et al., 2008). Steady-state ritonavir increased the AUC 0-∞ /dose ratio for intravenous and oral alfentanyl of 4 and 10folds, respectively, while reducing the hepatic extraction (0.26 to 0.07) and intestinal extraction (0.51 to zero) and increasing bioavailability (37 to 95%) in healthy volunteers. In total, 8 weeks of BID 400/100 mg of lopinavir/ritonavir in association wiht HIV-positive pregnant women resulted in the placental transfer of bupivacaine being 100% higher than in control pregnant women (Ribeiro et al., 2018).

Despite the small number of studies addressing the effects of the anesthetic agents in COVID patients, the specific risk of drug–drug interactions and side effects of COVID-19 medications have to be taken into account during the choice of the anesthesia technique. The opioid-sparing effect of RA can also be used in the preoperative management of this fragile group of patients (Jakobsson and Johnson, 2016).

## Regional Anesthesia and Coagulation Disorders

Thrombotic complications seem to emerge as an important issue in patients with COVID-19. Approximately 20% of the patients present severe coagulation abnormalities, and almost all patients with severe and critical COVID-19 infection showed major coagulation disorders with the risk of developing disseminated intravascular coagulation (CID) (Guan et al., 2020). In particular, preliminary reports on the characteristics of the COVID-19 disease have shown that infected patients commonly develop thrombocytopenia and elevated plasmatic D-dimer (Guan et al., 2020). Both thrombocytopenia and elevated D-dimer can be explained by the excessive activation of the coagulation cascade and platelets (Guan et al., 2020). Viral infection elicits the systemic inflammatory response and causes an imbalance between procoagulant and anticoagulant homeostatic mechanisms. Platelets are key mediators of inflammation and sensors of infectious agents through the interaction of cell surface receptors and pathogens or immune system derivatives. The activation of and interactions between macrophages, monocytes, endothelial cells, platelets, and lymphocytes play a critical role in the procoagulant effect of viral infection (Giannis et al., 2020).

The hypercoagulability state in patients affected by COVID-19 disease is supported by the results of the study by Panigada et al. (2020) (Panigada et al., 2020). The authors evaluated with thromboelastography (TEG) samples of whole blood from 24 patients admitted to the ICU because of COVID-19 pneumonia. TEG parameters were consistent with a state of hypercoagulability as shown by decreased values of R and K together with increased values of K angle and MA.

The coagulation abnormalities observed may explain the events of venous thromboembolism (VTE) described in some of these patients and support the indications of the antithrombotic prophylaxis/treatment. The current recommendations for VTE prevention in COVID-19 disease include the use of LMWH as a first-line treatment for all critically ill patients and for mild, moderate COVID positive patients with acute medical diseases. The dose of LMWH should be based on both patient body weight and the D-dimer value, ranging between 100 IU kg^−1^ to 150 IU kg^−1^ ^24^. As a consequence, many patients may require higher doses of anticoagulant and, consequently, an adequate protocol for interrupting and resuming therapy to allow for safe surgery and, whenever possible, RA.

Moreover, LMWH might be used systematically in the treatment of COVID-19 infection for its potential direct anti-viral effect in competing with cells in the interaction with the SARS-CoV-2 Spike S1 protein receptor-binding domain (Tang et al., 2020). Indications for the interruption/initiation of anticoagulant therapy with regards to neuraxial puncture and for deep peripheral nerve blocks should be well established when considering RA indication (Tables 2,3) (Leffert et al., 2018; Narouze et al., 2018).

**TABLE 2:** Time between last dose of Anticoagulants and puncture or catheter insertion or removal.

Anticougulant Regimen	Time between last dose of Anticoagulantsand puncture or catheter insertion or removal
Low Dose LMWH enoxaparin ≤ 40 mg SQ once daily or deltaparin 5000 U SQ once daily	≥12 h since last tase
Intermediate Dose LMWH enoxaparin > 40 mg SQ once daily or 30 mg SQ twice daily and < 1 mg/kg SQ twice daily or 1.5 mg/kg SQ once daily or deltaparin > 5000 U SQ once daily and <120 U/Kg SQ twice daily or 200 U/kg SQ once daily	Insufficient published data to recommend a specific interval between 12–24 h to delay neuraxial anesthesia
High Dose LMWH enoxaparin: 1 mg/kg SQ twice daily or 1.5 mg/kg SQ once daily or deltaparin: 120 U/kg SQ twice daily or 200 U/kg SQ once daily	≥ 24 h since last tase
UHF	PTT range
Fondaparinux	48–96 h*

**TABLE 3:** Time between the last dose of Direct Oral Anticoagulants (DOAC) and puncture or catheter insertion or removal. Peripheral Nerve Blocks (PNBs); NPs; (Neuraxial Procedures); Deep Peripheral Nerve Blocks (dPNBs).

	Dabigatran		Apixaban–Edoxaban–Rivaroxaban	
	PNBs^a^	NPs + dPNBs^b^	PNBs^a^	NPs + dPNBs^b^
CrCL ≥ 80 ml/min	≥24 h	≥48 h	≥ 24 h	≥ 48 h
CrCL 50-79 ml/min	≥36 h	≥72 h	≥ 24 h	≥ 48 h
CrCL 30-49 ml/min	≥48 h	≥ 96 h	≥24 h	≥ 48 h
CrCL 15-29 ml/min	Not indicated	Not indicated	≥36 h	≥ 48 h
CrCL < 15 ml/min	No official indication to use			

a except Deep Peripheral.

b Defines as blocks involving anatomical regions where a direct compression/heamostasis is possible and/or the potential haematoma will not involve neurological structures.

## Conclusion

Based on the potential advantages related to the application of RA techniques in both COVD-free and COVID-positive patients, we believe that RA is a fundamental weapon for the anesthetists in the COVID-19 age. Although, the management of a confirmed or suspected COVID-19 patients requires caution and the careful evaluation of both actual therapy and the coagulation state to prevent undesirable side effects.
